# Impact of Neuropathic Pain at the Population Level

**DOI:** 10.14740/jocmr1675w

**Published:** 2014-02-06

**Authors:** Ana Shirley Maranhao Vieira, Abrahao Fontes Baptista, Livia Mendes, Kamilla Soares Silva, Sharize Cristine de Araujo Gois, Flavia Manoela de Almeida Lima, Israel Souza, Katia Nunes Sa

**Affiliations:** a Escola Bahiana de Medicina e Saude Publica, Salvador, Bahia, Brazil; b Universidade Federal da Bahia, Salvador, Bahia, Brazil; c Instituto Federal de Educacao, Ciencia e Tecnologia do Rio de Janeiro, Rio de Janeiro, Brazil

**Keywords:** Pain, Chronic pain, Population, Quality of life, Neuropathic pain

## Abstract

**Background:**

One of the chief complaints of individuals who frequent the Family Health Units is chronic pain which, in Salvador, affects over 40% of the population. However, little is known about the type of pain and its impact on quality of life (QoL) at population level. The aim of the study is to evaluate the impact of neuropathic pain on QoL in a community.

**Methods:**

A descriptive cross-sectional study was conducted from March to October 2012, in a Family Health Unit, Salvador, Bahia, Brazil. The DN-4 (type of pain), body map (location), VAS (intensity) and SF-36 (QoL) instruments were applied. The Chi-square (univariate analysis) and logistic regression (multivariate) tests were used, with IC 95% and P < 0.05.

**Results:**

In a sample of 191 individuals with chronic pain, predominantly women (86.4%), single (48.7%), nonwhite (93.2%), low educational (46.6%) and low economic (100%) level. The most affected locations of the body were knees, lumbar region and head. In 60.2% of interviewees, neuropathic pain, of high intensity (VAS = 7.09 ± 3.0) predominated, with duration of 8.53 ± 8.8 years and mean QoL was reduced in 47.13%.

**Conclusions:**

Intense pain in the dorsal region and type of neuropathy are independent predictors for greater compromise of QoL.

## Introduction

Pain affects different aspects of human beings and its interpretation varies from one cultural and socioeconomic condition to another. It is a multifactorial phenomenon that involves everything from tissue damage to environmental aspects [1, 2]. According to IASP (International Association for the Study of Pain), pain is an unpleasant sensory and emotional experience associated with real or potential lesions of tissues, or described in their terms [3]. When the pain lasts for longer than 6 months, it is classified as chronic. It compromises the quality of life (QoL), making the individual unable to perform activities [1].

Chronic pain has been shown to be associated with the symptoms of neuropathic pain and this association affects QoL [4]. Neuropathic pain is defined as a result of injury nerve, and is regarded as a complex syndrome with organic mechanical yet fully understood [5].

In spite of being a public health problem in Brazil, little is known about chronic pain, especially at the population level [6]. It is essential to define the type of pain, intensity, site affected and its impact on the QoL.

The Family Health Units (FHUs) provide follow-up of persons with chronic diseases [7]. One of the most common complaints found in the communities is pain, which in Salvador, affects 41.4% of the population [8]. The Basic Health Care professionals are responsible for a person’s first contact with the health system. They may, however, contribute to tracing the causal factors of chronic pain and interfere in control of the problem [9].

The term QoL related to health has been implicated by the aspects most directly associated with the diseases, by including the perception of persons who suffer from the problem [10-12]. Different instruments are used for evaluating QoL and health status [11, 13]. Therefore, the aim of this study was to evaluate the impact of neuropathic pain on the QoL in a community.

## Methods

A descriptive, cross-sectional population-based study was conducted in the FHU Zulmira Barros, Salvador, Bahia, Brazil. The multiprofessional team that acts in the Unit serves the communities Recanto Feliz and Paraiso Azul. It comprises a population of 2,357 inhabitants, according to the Basic Care Information System/2011.

Individuals registered with the FHU, 20 years of age or older, who had been in pain for a period equal to or longer than 6 months, were selected. The criteria for exclusion from data collection were pregnant women, and individuals who were cognitively incapable of responding to the questionnaires.

After applying the inclusion and exclusion criteria, the population was reduced to 1,550 persons. When applying a prevalence of 41.4% of chronic pain in Salvador [8], the number of affected persons was estimated at 620 individuals. Calculating the sample according to WINPEPI (Package of Statistical Programs for Epidemiologists for Windows), with an interval of confidence of 95% and acceptable difference of 7%, the proposal was to conduct the study with 190 individuals.

The participants were approached in the FHU, or by indication and in the company of the community health agents, visited in their homes and were invited to participate in an interview with the application of questionnaires to characterize pain. All the participants signed the term of free and informed consent.

The instruments used were as follows: questionnaires on sociodemographic and clinical aspects, and versions validated for Brazilian Portuguese of DN-4 (questionnaire for diagnosis of neuropathic pain) [14] and of SF-36 (Short Form 36) [13]. The body map was also used to identify the sites affected, and the visual pain scale to measure the intensity.

The period for conducting the field research occurred during the months from March to October, 2012. In order to guarantee the quality of data, the same instruments were re-applied in 10% of the studied population, to evaluate the inter-examiner agreement, and after one month, the instrument was re-applied in the same proportion of the studied population to guarantee intra-examiner agreement.

### Statistical analyses

The independent variables were: sex, age, marital status, skin color, alcoholism, smoking, time, intensity, site and type of pain. The dependent variable was the impact on QoL of those affected by pain. The data obtained were tabled and analyzed in the Program SPSS for Windows version 17.0. For the inferences, the Chi-square test was used in the univariate analysis for nominal variables. To verify the independent impact of the studied variables on the type of pain and QoL, the logistic regression technique was used, which allows the odds ratio (OR) to be estimated from the medians, indicating which characteristic is implicated in greater or lower chance of leading to an “uncompromised” or “compromised” QoL. Initially, univariate logistic regression models were adjusted for all the explanatory variables, considering a level of significance of 10% (P < 0.10) for entry into the model.

The variables selected at this stage were introduced into a single model, and afterwards, the automatic stepwise backward selection procedure was performed, so that only those significant at 5% or those that contributed to better adjustment of the model remained in the model. Adjustment of the models was evaluated by the Hosmer-Lemeshow test.

In the specific case of the dimensions QoL measured by SF-36, these were dichotomized based on the median for use in the logistic regression. Individuals with a score equal to or below the median were classified has having a “compromised QoL”, and those with a score higher than the median, as having an “uncompromised QoL”.

This study was approved by the Ethics Committee of the Bahian School of Medicine and Public Health, and authorized by the Municipal Secretary of Health of Salvador.

## Results

The sample of this study was composed of 191 individuals. The mean age of the participants was 46.2 ± 13.8 years.

With reference to sociodemographic characterization, the study population was composed of 86.4% of the female sex, the majority being single, self-reported as being of nonwhite color. The majority of the sample consisted of nonsmokers and those who did not consume alcohol (Table 1).

**Table 1:** Sociodemographic Characterization of the Sample Population of a Registered Health Unit, Salvador, Bahia, Brazil

Variables		n	%
Sex	Female	165	86.4
	Male	26	13.6
Age	20-30	23	12.0
	31-60	144	75.4
	61-90	24	12.6
Marital status	Single	93	48.7
	Married	71	37.2
	Others	27	14.1
Skin color	Nonwhite	178	93.2
	White	13	6.8
Smoking	No	115	60.5
	Yes	29	15.3
	Ex-smoker	46	24.2
Consumption of alcoholic beverages	Non-consumer	114	59.7
	Moderate	72	37.7
	Excessive	5	2.6

**Figure 1:**
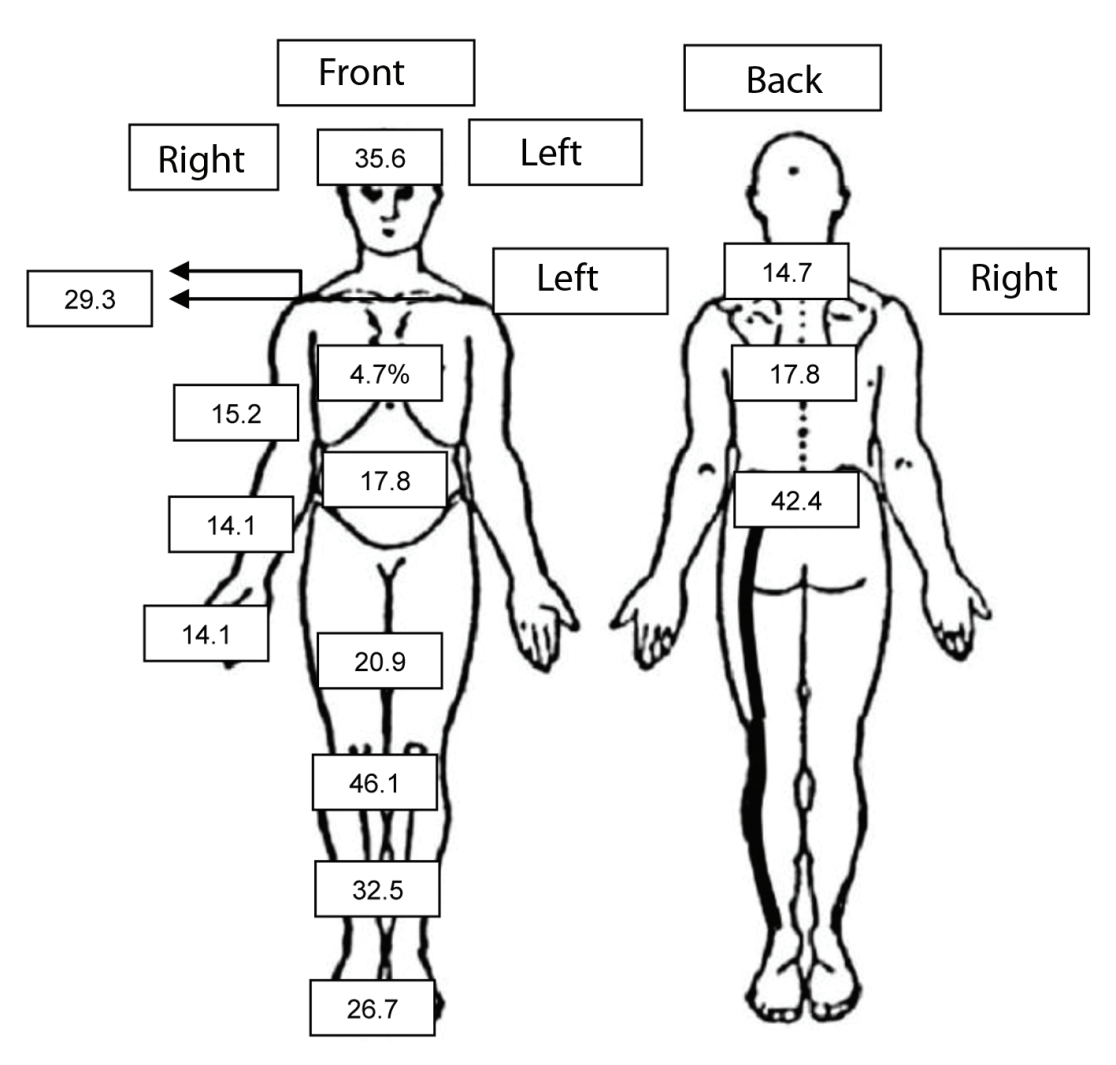
Sites in the body affected by pain in the population sample of a registered health unit, Salvador, Bahia, Brazil.

As regards the clinical characterization of pain, the individuals reported feeling pain for at least 5 years, and 25.7% reported intense pain. The sites in the body most affected by the presence of pain are shown in Figure 1. By means of DN-4, 60.2% of the subjects were classified as having neuropathic pain.

The intensity of pain in these individuals showed a mean of 7.09 ± 3.0, with mean duration of 8.53 ± 8.8 years. The general state of health was considered good by 64.4% of the sample.

The QoL was reduced in 47.13%, and Table 2 lists the most compromised domains.

**Table 2:** Impact of Pain on the QoL of the Population Sample of a Registered Health Unit, Salvador, Bahia, Brazil

QoL (SF-36 in %)	Median	Compromised QoL n (%)	Uncompromised QoL n (%)
Functional capacity	65.00	96 (50.26)	95 (49.74)
Physical aspect	50.00	106 (55.50)	85 (44.50)
Pain	45.00	97 (50.79)	94 (49.21)
General state of health	57.00	104 (54.45)	87 (45.55)
Vitality	60.00	98 (51.31)	93 (48.69)
Social aspect	62.50	112 (58.64)	79 (41.36)
Emotional aspect	33.33	97 (50.79)	94 (49.21)
Mental health	64.00	97 (50.79)	94 (49.21)
Total score	52.87	97 (50.79)	94 (49.21)

As there was higher prevalence of neuropathic pain, the option was taken to consider it as outcome for the regression model. For this, multiple logistic regression was performed, aiming to assess the impact of a single variable in “neuropathic pain”, in addition to confidence intervals of 95%.

When analyzing each variable alone, it was noted that at a level of significance lower than or equal to 10%, only the presences of pain in the shoulders, in the dorsal region, in the arms, in the hands, in the thighs, in the knees, in the legs and in feet were significant, always indicating that having pain in these locations increased the individual’s chance of being diagnosed with “neuropathic pain” (Table 3). Sociodemographic variables and habits of life presented no significant differences between the groups of “compromised” and “uncompromised” QoL.

**Table 3:** Multiple Logistic Regression Predictors of “Neuropathic Pain” in a Population Sample of a Health Unit, Salvador, Bahia, Brazil

Variables	B	Standard error	Wald	df	P	Crude OR (IC 95%)
Pain in shoulders	0.935	0.354	6.994	1	0.008	2.548 (1.274 - 5.096)
Constant	0.163	0.173	0.894	1	0.344	1.177
Pain in dorsal region	1.323	0.478	7.672	1	0.006	3.755 (1.472 - 9.576)
Constant	0.217	0.161	1.834	1	0.176	1.243
Pain in arms	1.070	0.485	4.870	1	0.027	2.917 (1.127 - 7.547)
Constant	0.273	0.159	2.969	1	0.085	1.314
Pain in hands	1.504	0.564	7.109	1	0.008	4.500 (1.489 - 13.596)
Constant	0.245	0.157	2.427	1	0.119	1.278
Pain in thighs	1.187	0.428	7.699	1	0.006	3.277 (1.417 - 7.579)
Constant	0.199	0.164	1.485	1	0.223	1.221
Pain in knees	0.718	0.304	5.580	1	0.018	2.050 (1.130 - 3.719)
Constant	0.097	0.197	0.243	1	0.622	1.102
Pain in legs	0.692	0.331	4.364	1	0.037	1.997 (1.044 - 3.821)
Constant	0.202	0.177	1.306	1	0.253	1.224
Pain in feet	1.625	0.421	14.924	1	< 0.001	5.076 (2.226 - 11.575)
Constant	0.057	0.169	0.114	1	0.735	1.059

All the variables that were significant (P < 0.10) entered into the final modeling (Table 4).

**Table 4:** Multivariate Analysis Adjusted for Independent Predictors of Neuropathic Pain in the Population Sample of a Registered Health Unit, Salvador, Bahia, Brazil

Variables	B	Standard error	Wald	P	Adjusted OR (IC 95%)
Pain in dorsal region	1.355	0.503	7.261	0.007	3.879 (1.447 - 10.395)
Pain in hands	1.236	0.597	4.283	0.038	3.440 (1.068 - 11.085)
Pain in thighs	0.974	0.460	4.488	0.034	2.649 (1.076 - 6.522)
Pain in feet	1.378	0.442	9.730	0.002	3.969 (1.669 - 9.437)
Constant	-0.393	0.206	3.657	0.056	0.675

χ^2^ = 36.943; gl = 4; P < 0.001.

In the final model, the presences of pain in the dorsal region, in the hands, in the thighs and in the feet were maintained as independent predictive variables for neuropathic pain. After the Hosmer-Lemeshow statistics (χ^2^ = 0.196; P = 0.995), it was able to correctly classify 68.6% of the individuals.

All these procedures were adopted in each of the domains of SF-36; however, the option was taken to present only the main findings by means of texts, due to the limit on tables and pages of the article.

In the analysis of impacts on the emotional aspects of QoL in patients with chronic pain, the final model maintained the variables neuropathic pain (P = 0.028) and presence of pain in the neck (P = 0.044) and thighs (P = 0.034) as independent predictive variables for compromised QoL, with reference to emotional aspects. The model presented adjustment criteria that indicated its adjustment both by the traditional criteria (P < 0.001), and in agreement with the Hosmer-Lemeshow statistics (χ^2^ = 1.625; P = 0.654), it was able to correctly classify 61.8% of the individuals.

With regard to the physical aspects of the instrument, the final adjusted model maintained the variables smoking (P = 0.041), pain in the dorsal region (P = 0.018) and neuropathic pain (P = 0.017) as independent predictors; that is to say, they diminished the individual’s chance of being classified as having an uncompromised QoL, with reference to physical aspects. The model presented adjustment criteria that indicated its adjustment both by the traditional criteria (P < 0.001), and in agreement with the Hosmer-Lemeshow statistics (χ^2^ = 2.292; P = 0.807), it was able to correctly classify 65.4% of the individuals.

As regards the impact on social aspects, the final model maintained the variables pain in the hands, in the neck and neuropathic pain. The presence of these pains diminished the individual’s chance of being classified as having an uncompromised QoL as regards social aspects. However, only the variable neuropathic pain was shown to be significant at the level of 5%, the others presented P > 0.05, but contributed to adjustment of the model. The model presented adjustment criteria that indicated its adjustment both by the traditional criteria (P < 0.001), and in agreement with the Hosmer-Lemeshow statistics (χ^2^ = 0.185; P = 0.980), it was able to correctly classify 64.4% of the individuals.

For the functional capacity domain, individuals between 31 and 60 years of age (P = 0.039) have less chance of being classified as having an uncompromised QoL, as regards functional capacity, and this was affected by the presence of neuropathic pain (P < 0.001) and pain in the dorsal region (P = 0.030). The model presented adjustment criteria that indicated its adjustment both by the traditional criteria (P < 0.001), and in agreement with the Hosmer-Lemeshow statistics (χ^2^ = 10.456; P = 0.234), it was able to correctly classify 67.5% of the individuals.

As regards the impact on the SF-36 domain of pain, after the adjusted analysis, the following variables of the raw analysis remained in the final model: time of pain between 5 and 10 years (P = 0.05) and pain in the dorsal region (P = 0.015), being variables that reduced the QoL with reference to the aspect of pain. The model presented adjustment criteria that indicated its adjustment both by the traditional criteria (P < 0.001), and in agreement with the Hosmer-Lemeshow statistics (χ^2^ = 1.376; P = 0.967), it was able to correctly classify 64.4% of the individuals.

In the domain of general state of health, the final model maintained the variables intense pain (P < 0.001) and type of neuropathy (P = 0.003) as independent predictors; that is to say, variables that diminished the individual’s chance of being classified as having an uncompromised QoL, with regard to the general state of health. The model presented adjustment criteria that indicated its adjustment both by the traditional criteria (P < 0.001), and in agreement with the Hosmer-Lemeshow statistics (χ^2^ = 4.901; P = 0.672), it was able to correctly classify 68.1% of the individuals.

In the domain of mental health, the final model maintained the variables intense pain (P = 0.008), pain in the head/headache (P = 0.002), pain in the neck (P = 0.014) and the type of neuropathy (P = 0.003) as independent predictors; that is to say, variables that diminished the individual’s chance of being classified as having an uncompromised QoL, with regard to the SF-36 domain of mental health. The model presented adjustment criteria that indicated its adjustment both by the traditional criteria (P < 0.001), and in agreement with the Hosmer-Lemeshow statistics (χ^2^ = 1.310; P = 0.995), it was able to correctly classify 68.6% of the individuals.

With regard to vitality, the final model maintained the variables time of pain exceeding 10 years (P = 0.018), and pain of the neuropathic type (P = 0.002), as independent predictors; that is to say, variables that diminished the individual’s chance of being classified as having an uncompromised QoL, with regard to the SF-36 domain of vitality. The model presented adjustment criteria that indicated its adjustment both by the traditional criteria (P < 0.002), and in agreement with the Hosmer-Lemeshow statistics (χ^2^ = 0.775; P = 0.942), it was able to correctly classify 61.8% of the individuals.

To assess the impact of a single isolated variable in “SF-36”, multiple logistic regression was performed, searching for the raw OR values, beyond the confidence intervals of 95%.

When analyzing each variable in isolation, it was noted that at a level of significance lower than or equal to 10%, having intense pain, in the shoulder, dorsal region, in the arms, of the neuropathic type (P < 0.001), diminished the individual’s chance of being classified as having an uncompromised QoL SF-36 (Table 5).

**Table 5:** Multiple Logistic Regression Predictors of the Total Score of SF-36 in a Population Sample of a Health Unit, Salvador, Bahia, Brazil

Variables	B	Standard error	Wald	df	P	Crude OR (IC 95%)
Severe pain	-0.785	0.358	4.797	1	0.029	0.456 (0.226 - 0.921)	
Constant	0.241	0.201	1.433	1	0.231	1.273	
Pain in shoulders	-0.890	0.331	7.217	1	0.007	0.411 (0.215 - 0.786)	
Constant	0.223	0.173	1.660	1	0.198	1.250	
Pain in dorsal region	-1.188	0.420	7.979	1	0.005	0.305 (0.134 - 0.695)	
Constant	0.166	0.160	1.074	1	0.300	1.181	
Pain in arms	-0.716	0.421	2.890	1	0.089	0.489 (0.214 - 1.116)	
Constant	0.074	0.157	0.222	1	0.637	1.077	
DN4 score (neuropathic pain)	-1.326	0.314	17.867	1	< 0.001	0.266 (0.144 - 0.491)

Constant	0.773	0.247	9.817	1	0.002	2.167	

The final model adjusted in this method may be observed in Table 6.

**Table 6:** Adjusted Analysis of the Predictors of the Total Score of SF-36 in the Population Sample of a Registered Health Unit, Salvador, Bahia, Brazil

Variables	B	Standard error	Wald	P	Adjusted OR (IC 95%)
Pain in dorsal region	-1.169	0.470	6.185	0.013	0.311 (0.124 - 0.781)
Time of pain less than 5 years			5.119	0.077	
Time of pain between 5 and 10 years	-0.403	0.375	1.152	0.283	0.668 (0.320 - 1.395)
Time of pain exceeding 10 years	0.626	0.422	2.203	0.138	1.870 (0.818 - 4.272)
Without pain at the moment			7.249	0.064	
Slight pain	-0.755	0.649	1.353	0.245	0.470 (0.132 - 1.677)
Moderate pain	-0.541	0.471	1.320	0.251	0.582 (0.231 - 1.466)
Severe pain	-1.048	0.401	6.814	0.009	0.351 (0.160 - 0.770)
DN4 score (neuropathic pain)	-1.227	0.333	13.563	< 0.001	0.293 (0.153 - 0.563)
Constant	1.294	0.360	12.936	0.000	3.649

χ^2^ = 36.355; gl = 7; P < 0.001.

The final model maintained the variables pain in the dorsal region, intense pain and type of neuropathy, as predictive variables for compromised QoL. The model presented adjustment criteria that indicated its adjustment both by the traditional criteria and in agreement with the Hosmer-Lemeshow statistics (χ^2^ = 9.186; P = 0.327), it was able to correctly classify 64.9% of the individuals.

## Discussion

In this study, it was observed that neuropathic pain caused the greatest repercussions in all the domains evaluated, which reinforces the importance of identifying neuropathic pain at population level.

In the studied community, women who were single, nonwhite, non-consumers of alcohol and nonsmokers made up the sociodemographic profile of the persons who suffer from chronic pain. Similar results have also been found in the literature [14-17].

In the raw descriptive analysis, it was observed that the sites most affected were the lumbar spine, knees and head; however, none of these sites was an independent predictor of neuropathic pain. The lumbar region has been the one most affected in various epidemiologic studies that evaluated the presence of chronic pain [18, 19]. Therefore, it was possible to identify that the lumber region, lower and upper limbs were the regions that caused the greatest repercussions on the loss of QoL when associated with neuropathic pain. It has been verified that neuropathic pain generally affects more than one site, and in the majority of cases, involves the region of the back and lower limbs [15, 20].

The participants presented moderate to intense pain, which is in agreement with that which has been observed in population studies when the prevalent type of pain is neuropathic [14, 21]. This datum points out the need for analgesic support in the FHUs, in addition to preventive actions. The intense pain was also strongly associated with neuropathic pain, and with the worst QoL indices in the multivariate analysis. In spite of the intense pain, the majority of the individuals declared that their general state of health was good. Phenomena such as religion, resilience and greater capacity to face up to pain in populations with a low socioeconomic level may have influenced this perception of the general state of health [20].

Individuals with neuropathic pain had their QoL reduced in all the domains. This finding was also confirmed, particularly in the physical and mental aspects [12]. The most compromised domains in the evaluation of QoL were the physical aspect, pain and the emotional aspect. These data confirm the findings of a study conducted by other researchers with individuals who presented the same domains, in the same order of compromise [22, 23]. When individuals are affected by chronic disease, it is not only the pathologic factor that influences the situation, because there are changes in their lives related to physical discomfort, losses of a personal, financial and social order [22]. It has been shown that the repercussion of chronic pain on the QoL is more related to neuropathic pain than to intensity or duration [20].

As regards the emotional aspects, the low scores in neuropathic pain have been confirmed by population studies [20]. In other studies, it has been observed that for the physical aspects, greater relationship has been found with smoking and pain in the region of the back [15]. Whereas for the domain of social aspects, our data point to the neck and upper limbs being more affected, but no similar data were found in the different studies. One supposes that the loss of functionality of the upper limbs related to the presence of neuropathic pain would impede those who are affected from continued participation in intense work and social activity.

When we sought variables that predicted compromised QoL in the individual with chronic pain, we found that having intense pain of the neuropathic type in the dorsal region reduced the individual’s chance of having a high QoL. All the QoL domains are harmed to a greater extent in individuals who report chronic pain with neuropathic characteristics [20]. Individuals that had chronic pain with neuropathic characteristics presented low scores in all the domains when compared with the scores presented by patients with other serious somatic diseases, as in the case of cardiopathies [14, 23].

### Conclusions

Cross-sectional studies have limitations inherent to the model, and this is why the results must take into consideration the difficulty of establishing causal relationships.

With this epidemiologic study, it was possible to dimension the impact of pain of the neuropathic type in a community with a low socioeconomic condition with an important reduction in the QoL of those in pain. These findings point out the need for pain prevention. In this low income population, it was observed that chronic pain mainly affected the regions of the knee and lumbar spine, presented high intensity, long duration and impact on different aspects of the QoL, irrespective of the sites affected. Women are affected to a greater extent, and neuropathic pain causes greater impact on the QoL than nociceptive pain does. It was therefore concluded that intense pain in the dorsal region and of the neuropathic type are independent predictors for greater compromise of QoL.
